# Supplementary material for: A Generalizable Multimodal Scrub Training Curriculum in Surgical Sterile Technique
Source: MedEdPORTAL. 2021 Feb 1;17:11077. doi: 10.15766/mep_2374-8265.11077 (PMC7852343; doi:10.15766/mep_2374-8265.11077)
Supplement: Supplementary file 1 — Instructor Guide.docxScrub Training Video.mp4Student Instructional Letter Template.docxScrub Training Knowledge Test.docxScrub Training Skills Checklist.docxScrub Training Pre- and Postsession Survey.docx [file mep_2374-8265.11077-s001.zip › _Educational_Summary_Report_11077.pdf]

MedEdPORTAL® The AAMC Journal of Teaching and Learning Resources

Original Publication

Open Access

A Generalizable Multimodal Scrub Training Curriculum in Surgical Sterile Technique

Tiffany N. Anderson, MD, MHPE*, Brittany N. Hasty, MD, MHPE, Ingrid S. Schmiederer, MD, Sarah E. Miller, MD, Robert Shi, MS, Lauren R. Aalami, Elizabeth M. Huffman, MD, Jennifer N. Choi, MD, James N. Lau, MD, MHPE

*Corresponding author: TNAnders87@gmail.com

Abstract

Introduction: Recent endeavors from governing bodies such as the AAMC have formally recognized the importance of aseptic technique. AAMC guidelines include activities that all graduating physicians should be able to perform with minimum indirect supervision and were developed to recognize these needs. For example, the skills necessary for aseptic technique include daily safety habits and general physician procedures. **Methods:** We developed a scrub training curriculum and evaluated the program through a quasi-experimental study with a pre- and posttest design. Questions were developed to examine students' perceived knowledge and skills as related to the objectives of the course and to their anxieties, concerns, and future training needs. **Results:** Between February 2020 and March 2020, 44 students completed the curriculum. Students indicated that self-efficacy significantly increased in all aspects of the curricular goals following curriculum completion. Students identified understanding OR etiquette as the most anxiety-provoking element associated with scrub training. They felt that more time could be spent elucidating this etiquette. On the other hand, tasks such as surgical hand hygiene were the least anxiety-inducing. **Discussion:** We share this multimodal scrub training curriculum, mapped to the AAMC's guidelines, to reduce variability in teaching strategies and skills acquisition through a standardized curriculum. Also, we effectively imparted these skills and instilled a sense of confidence in learners as they worked to provide their best in patient care and safety.

Keywords
Sterile Technique, Aseptic Technique, Scrub Training, Operating Room Etiquette, In Situ Simulation

Educational Objectives

By the end of this activity, learners will be able to:

1. Describe appropriate surgery attire for OR personnel.
2. Identify all personal protection equipment necessary for entry into the OR suite.
3. Demonstrate effectively and independently proper surgical hygiene, donning of a mask, gowning, and gloving.

Introduction

Historically, there have been concerns that the basic principles of aseptic technique were not prioritized in medical training, though recent endeavors from governing bodies such as the AAMC have formally recognized the importance of aseptic technique.[1] Two of the 13 AAMC Core Entrustable Professional Activities (EPAs)—activities that all graduating physicians should be able to perform with minimum indirect supervision—were developed to recognize these needs. Specifically, EPAs 12 and 13 apply to the skills necessary for aseptic technique such as, "engage in daily safety habits," and the ability to "perform general procedures of a physician."[2,3]

Studies show that students regarded the ability to appropriately perform the skill of scrubbing in was essential to OR-based learning.[4-6] Despite this, a majority of students report no formal introduction to the scrub process prior to participating in the OR.[6-8] *Scrubbing in* is a colloquial term, describing the act of washing one's fingernails, hands, forearms, and elbows with a bactericidal soap or solution in a methodical manner prior to the start of any surgical procedure. Appropriate preprocedural hand washing has been proven to improve patient care. Inappropriate techniques can lead to adverse patient outcomes, such as surgical site infections, which may increase patients' length of hospitalization, cost of care, and risk of mortality.[9]

Citation:
Anderson TN, Hasty BN, Schmiederer IS, et al. A generalizable multimodal scrub training curriculum in surgical sterile technique. *MedEdPORTAL.* 2021;17:11077.
https://doi.org/10.15766/mep_2374-8265.11077

Copyright © 2021 Anderson et al. This is an open-access publication distributed under the terms of the Creative Commons Attribution license.

An understanding of sterile technique is necessary for participation in surgical clinical activities. Previous studies have evaluated surgical skills curricula with a scrub training component and have found that students' confidence in performing these techniques increased, and anxiety decreased, after these scrub training sessions.[10-12] It was our intent to reduce variability in teaching strategies and skills acquisition through a standardized curriculum. We designed an EPA-aligned multimodal curriculum that first began in 2015 and has been proven to increase both student knowledge of and confidence in scrub training.[13] We share our program's most recent iteration which offered an efficient and immersive in situ training experience and influenced learners' perceived self-efficacy in donning OR attire, utilizing scrub training techniques, and acting in accordance with OR etiquette.

Methods

Participants and Setting

Medical specialty students—medical (MD), physician assistant (PA), and dental (DMD)—at our institution undergo 1 to 2 years of preclinical study followed by 2 to 3 years of clinical (clerkship) experience. Prior to participating in the OR or entering their clinical years (which include surgical and surgical subspecialty rotations), students are required to undergo formal instruction on aseptic technique and OR etiquette. Recently, newly implemented programs at our institution designed to encourage early surgical exposure have motivated many preclinical students to seek scrub training in their first year.[10] Over 600 students have participated in this iteration of the scrub training course from July 2018 to March 2020. We re-evaluated the course sessions during the months of February 2020 and March 2020 as elements of the course (such as instructors, timing, and location) had remained mostly consistent for a 12-month stretch. The instructors consisted of the same surgical attending, surgical residents, and OR nurse educator. Due to pending institutional reduction of onsite educational activities secondary to global health-related concerns, our evaluation period was abbreviated to the presented period.

Scrub training course sessions were conducted one to two times per month based on clerkship rotation schedule and leadership-directed need. Sessions occurred on Mondays during which operations/endoscopies/interventional procedures started 1 hour later. This allowed for the use of the OR suite for in situ training. Students were asked to present at 6 am for check-in and instructor assignment (approximately 15 minutes). Class sizes typically ranged from eight to 40 students. The student-to-teacher ratio was designed to not exceed eight to one. OR space was requested that morning as availability was largely dependent upon planned procedures. All efforts were made to have an adequate number of OR rooms such that there were no more than 25 students to a given room. The facilitated practice sessions spanned the course of 60-90 minutes depending on class size and learners' needs. The final 10-15 minutes of the sessions were reserved for clean up and distribution of the students' scrub stickers, a marker of course completion.

A brief outline of a typical in situ scrub training day is provided in Figure 1.

Equipment

Each session required that all students possessed:

- Institutional specific OR clothing (scrubs)
- 1 disposable surgical cap (bouffant, surgeons cap, or beard cap)
- 1 surgical mask with attached visor for eye protection
- 1 sterile surgical gown in sterile wrapping
- 1 pair of size appropriate surgical gloves

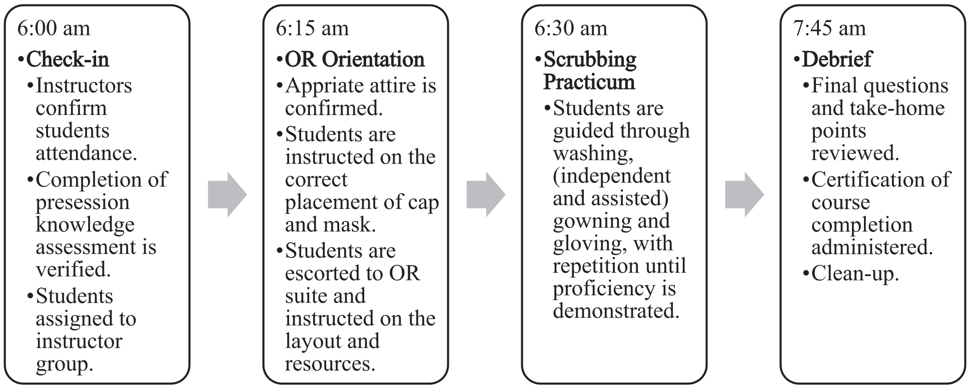

Figure 1. In situ scrub training day timelines.

Copyright © 2021 Anderson et al. This is an open-access publication distributed under the terms of the Creative Commons Attribution license.

- 1 pair of size appropriate indicator surgical gloves
- 1 surgical scrub brush
- 1 sterile hand towel
- OR tables to allow at least 2.5 meters squared of surface area for each learner
- Appropriate waste disposal receptacles

These materials were obtained as donations by the hospital, often due to expiration or sterility infraction of the product (Appendix A).

Curriculum

This multimodal curriculum consisted of two overall phases. First, students were required to watch an 18-minute video, created in alignment with the four learning objectives identified in a prior needs assessment for entry-level aseptic technique (Appendices B and C).[1,5] Students were encouraged to rewatch the video—made remotely viewable—to enable flexible, self-paced learning. Students were then asked to complete a postvideo knowledge assessment (Appendix D; the product of an iterative design process) with a required passing score of 92%. The final cut score was established using the Angoff method and adapted to adhere to Mastery Learning standards. The validity evidence for the knowledge assessment, as previously described, can be found in Hasty et al.[13]

Students then attended an in-person practical scrub training session lead by OR nurse educators, surgical education residents, and faculty surgeons (who had been previously trained as evaluators—see Appendix A). Prior to the hands-on activities, instructors administered an 11-item presession self-efficacy (SE) survey (described below). The students then engaged in deliberate practice—with feedback from the aforementioned instructors—until they were able to perform the steps of the surgical scrub without contamination.[14] A skills task cognitive aid (which could also be used for assessment purposes) was available for each session (Appendix E). Students were then asked to complete a 13-item postsession SE assessment that contained three free response program evaluation questions (described below). Students who passed both the knowledge assessment and in-person practicum were granted scrub privileges for indirect supervision while scrubbing, signified by stickers on their student badges for easy identification.

The curriculum and all materials were reviewed independently by nonaffiliated individuals for feasibility and generalizability (authors Elizabeth M. Huffman and Jennifer N. Choi). They were selected as peer reviewers as individuals from an academic institution that encompassed the largest medical training program and physician network in their state. Both have extensive experience as educators to aseptic technique and practical experience as surgeons.

Evaluation Strategy and Data Collection

We evaluated our program through a quasi-experimental study with a pre- and postsurvey design. Eight questions were developed that examined each student's perceived knowledge and skills ability as related to the objectives of the course. A SE method was chosen, as perceived efficacy (the ability to do) can be corroborated with objective measurements in a manner not feasible through confidence assessments.[15] The SE questions utilized a 5-point scale (1 = *not able*, 2 = *poor*, 3 = *fair*, 4 = *moderate*, 5 = *excellent*). In addition to the eight SE questions, the presession survey (Appendix F) also contained two questions related to students' personal experience with formal scrub training, and both the pre- and postsession surveys (Appendix F) contained three free response questions to determine how we might assist students in future iterations of the program. The surveys contained no identifying features and were matched using randomly generated numbers assigned to each student.

As this is a required session for all students that may participate in the OR, demographic data such as medical training program (MD, PA, or DMD) and year in school were collected but not matched to the survey results.

Data Analysis

Data were stored on a statistics software platform, Excel (Microsoft). Descriptive statistics and paired *t* tests were used to analyze the self-efficacy assessments. Free responses were categorized to domains corresponding to the different learning objectives and frequency of occurrence were counted. Counts were compared using two factor analysis of variance comparing responses from students with no prior scrub training (inexperienced) to those with formal experience (experienced), the qualitative data was reviewed by two reviewers (authors Tiffany N. Anderson and Robert Shi). Significance was determined at $p \leq .05$.

Ethical Approval

This study was approved as an exempt protocol by the Stanford University Institutional Review Board.

Results

Demographics

Between February and March 2020, a total of 44 students participated in the scrub training curriculum. The pre- and postsession SE assessment surveys were distributed to each

Copyright © 2021 Anderson et al. This is an open-access publication distributed under the terms of the Creative Commons Attribution license.

student. The response rate for the pre- and postsession surveys was 95% (42 out of 44). The results from two participants' surveys were missing their paired counterpart, so were not included in the analysis. Of students who participated, 27 were clerkship PA students, 15 were third-year clerkship MD students, and two were clerkship DMD students. Of students who completed the pre-/postsession SE survey, 22 had prior scrub training experience (52%). All of these students also possessed at least one intraoperative experience, so were considered experienced students for data reporting. Of the students who did not have formal scrub training ($n = 20$), none had intraoperative experience; we referred to them as inexperienced students (Table 1).

Table 1. Participant Characteristics

Demographics	Number (%)
Students ($N = 44$)[a]	
Dental medical degree program	2 (5)
Medical degree program	15 (34)
Physician assistant degree program	27 (61)
Experience ($N = 42$)[a]	
Previous scrub training	
Yes	22 (52)
No	20 (48)
Operative participation	
Yes	22 (52)
No	20 (48)
Scrub training and operative participation (experienced)	
Yes	22 (52)
No	20 (48)
Scrub training and no operative participation (inexperienced)	
Yes	
No	42 (100)

[a] All students were on their clinical (clerkship) rotations. Of the 44 who participated, only 42 submitted complete pre-/postsurveys.

Pre-/Postsession Survey SE Questions

For all eight questions, there was a statistically significant difference for each pre-/postsession SE survey response (Figure 2). There was no difference between experienced students' and inexperienced students' presession assessments. There was a statistically significant difference between experienced and inexperienced students' postsession assessments of mean SE (4.7, $SD = 0.2$, and 4.4, $SD = 0.3$, respectively, $F = 41.50$, $F_{crit} = 5.30$, $p = .0002$). Of note, one student answered that they could not perform the activity in question 7 of the pre- and postsession survey.

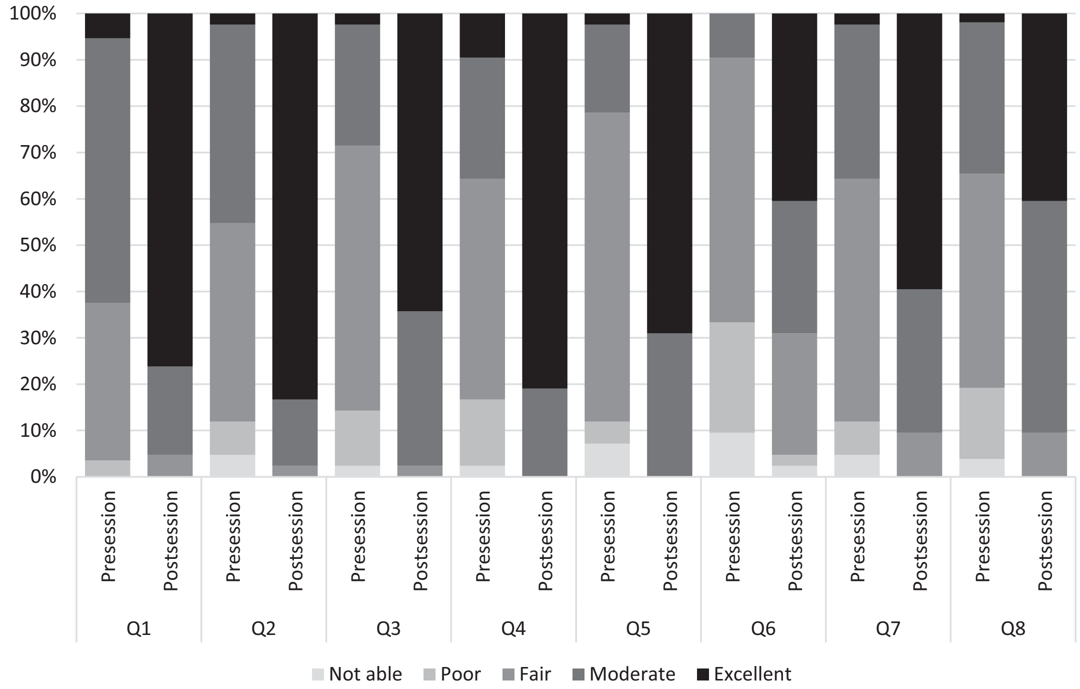

Figure 2. Pre/postsession perceived self-efficacy for students ($N = 42$), where all changes in pre- to postsession values were statistically significant ($p < .001$). Abbreviations: Q1, What is your current ability to correctly describe appropriate attire OR personnel?; Q2, What is your current ability to correctly identify all personal protection equipment necessary for entry into the OR suite?; Q3, What is your ability to appropriately perform a surgical hygiene scrub?; Q4, What is your ability to select and don the appropriate mask?; Q5, What is your ability to perform the assisted gowning and gloving technique without contamination?; Q6, What is your ability to perform the independent gowning and gloving technique without contamination?; Q7, What is your ability to identify sterile versus non-sterile surfaces within the operating suite?; Q8, What is your ability to identify sources of contamination within the operating suite?.

Copyright © 2021 Anderson et al. This is an open-access publication distributed under the terms of the Creative Commons Attribution license.

Pre-/Postsession Survey Free-Response Questions

When asked "What aspect of the scrubbing-in process causes you the MOST anxiety?", activities that arose in both the experienced and inexperienced groups of students statistically showed no difference ($F = 1.62$, $F_{crit} = 5.12$, $p = .23$). However, there was a significant difference between the numbers of times the students reported those activities ($F = 6.61$, $F_{crit} = 3.17$, $p = .005$). The activities most frequently cited as anxiety-provoking by both groups of students on pre- and postsession surveys were the practice of gloving (49% of students; Table 2) and OR etiquette (39% of students). Interestingly, more students in the experienced group cited the washing/scrubbing process as concerning, as compared to the inexperienced group: six and two students, respectively.

When asked "What aspect of the scrubbing-in process causes you the LEAST anxiety?", activities that arose in both the experienced and inexperienced groups of students statistically showed no difference ($F = 0.45$, $F_{crit} = 6.61$, $p = .53$). However, there was a significant difference between the numbers of times the students reported those activities ($F = 16.50$, $F_{crit} = 5.05$, $p = .004$). The activities most frequently cited as the least anxiety-provoking by both groups of students on pre- and postsession surveys were the practice of washing/scrubbing (61% of students) and gowning (36% of students).

When asked "What aspect of the scrubbing-in process should we spend more time on?", activities that arose in both the experienced and inexperienced groups of students showed no statistical difference ($F = 0.26$, $F_{crit} = 5.59$, $p = .63$). However, there was a significant difference between the numbers of times the students reported those activities ($F = 9.30$, $F_{crit} = 3.79$, $p = .004$). Based on pre- and postsession surveys, the activities students were most interested in spending more time on were the discussion of OR etiquette (36% of students), orientation (21% of students), and gowning (20% of students, with an emphasis on independent technique) for both groups. Four experienced students felt as though their experience was sufficient and that there was improvement since they last took the course, which we categorized as nothing/other.

Discussion

To our knowledge, this was the first multimodal scrub training curriculum created to partially address two of the core AAMC EPAs[2,3] focused on patient safety which also incorporated a knowledge assessment supported by robust validity evidence and facilitated deliberate practice. Although this curriculum was tailored for the operative theater, many of the principles and concepts taught were applicable to a number of situations requiring aseptic technique. We have found that our learners gain a greater sense of ability to perform this activity after undergoing all aspects of the curriculum. The presession assessment prompted students to pass personal judgement on their capabilities. Within the practicum session, this judgement was tested and refined through feedback-guided practice,

Table 2. Ranking of Student Responses to Pre- and Postsession Survey Free-Response Questions

Question	Ranked Response	Presession		Postsession		
		Experienced No. (%)[a]	Inexperienced No. (%)[b]	Experienced No. (%)[a]	Inexperienced No. (%)[b]	Pre- & Postsession Total No. (%)[c]
What aspect of the "scrubbing-in" process causes you the MOST anxiety?	Gloving	13 (59)	7 (35)	13 (59)	8 (40)	41 (49)
	OR etiquette	10 (45)	6 (30)	11 (50)	6 (30)	33 (39)
	Washing/scrubbing	6 (27)	2 (10)	6 (27)	2 (10)	16 (19)
	Gowning	3 (14)	4 (20)	3 (14)	5 (25)	15 (18)
	Sterility	3 (14)	4 (20)	2 (9)	4 (20)	13 (15)
What aspect of the "scrubbing-in" process causes you the LEAST anxiety?	Washing/scrubbing	14 (64)	12 (60)	14 (64)	11 (55)	51 (61)
	Gowning	6 (27)	9 (45)	7 (32)	8 (40)	30 (36)
	Gloving	3 (14)	1 (5)	2 (9)	1 (5)	7 (8)
	Orientation	3 (14)		3 (14)		6 (7)
					1 (5)	1 (1)
What aspect of the "scrubbing-in" process should we spend more time on?	OR etiquette	8 (36)	7 (35)	8 (36)	7 (35)	30 (36)
	Orientation	4 (18)	5 (25)	4 (18)	5 (25)	18 (21)
	Gowning	6 (27)	4 (20)	3 (14)	4 (20)	17 (20)
	Gloving	1 (5)	3 (15)	2 (9)	5 (25)	11 (13)
	Nothing/other	4 (18)		4 (18)	2 (10)	10 (12)
	Washing/scrubbing	2 (9)	3 (15)	2 (9)	3 (15)	10 (12)
	Organization/preparation		3 (15)	1 (5)	2 (10)	6 (7)
	Patient positioning	1 (5)				1 (1)

Abbreviation: OR, operating room.
[a]$n = 22$.
[b]$n = 20$.
[c]Percentage calculated out of total number of pre- and postsession survey responses (84).

Copyright © 2021 Anderson et al. This is an open-access publication distributed under the terms of the Creative Commons Attribution license.

and a new assessment of capabilities was created. Ideally, learners gained insight into their deficits and sought to rectify these areas. Although, we have elected to formally evaluate the program for this abbreviated time span, over the years and many sessions we have informally collected feedback from learners. These suggestions have informed the curriculum as has been presented.

We elected to share the concerns identified by the students after having participated in this curriculum by way of the free response questions. It was interesting to note that anxieties, comforts, and desires between the two types of learners were not significantly different given that all of the students who previously had taken the scrub training also had real world operative experience. Chief among the concerns for both groups was the acquisition of OR etiquette and adjustment to OR culture. Given the complex nature of the environment, this was likely a skill that was not amenable to a single introductory course. As the responses from the experienced students would suggest, it may also be an aspect of surgical culture that takes longer to fully absorb. Nevertheless, when we examined the students' reported efficacy in activities adherent to OR etiquette, there was a significant increase, which hopefully provided a foundation for learners to build upon. This same concept went for other items that students cited, such as gowning (assisted and independent), gloving (assisted and independent), and washing. However, these suggestions will help us to further tailor the experience to students' needs.

Although we teach learners how to perform both assisted and independent gowning and gloving, we spent proportionately more time on assisted technique with a focus on maintaining sterility as this is the most common technique they perform at our institution. We have also, on occasion, used portions of this curriculum (e.g., the video/knowledge assessment and one-on-one mentoring in an in vitro setting) to teach basic aseptic technique (washing and gloving, without gowning). We have at times provided the video/knowledge assessment alone to select individuals (who have taken the practical session within one year) who sought a basic refresher. These different approaches to the curriculum have been met with anecdotal satisfaction from the learners.

There were a few potential limitations with the generalizability of this curriculum. This course was conducted at single private academic medical institution. Therefore, our results may reflect different densities in knowledge of scrub training techniques and principles among US health professional trainees. In addition, our scrub training curriculum in its current form has thus far only been implemented for approximately 2 years, so long-term retention of the skills taught in this curriculum have yet to be assessed. Third, all instructors in this program were involved in the development of the scrub training curriculum and did not require more than 30 minutes of rater training to evaluate student scrub training technique. Potential instructors without previous exposure to our curriculum may require more rater training. There may also be a challenge for institutions that may not necessarily have the infrastructure to accommodate in situ practice sessions in an OR or true-to-life simulated environment. Although we have not yet studied our scrub training curriculum as it related to patient outcomes, we will continue to revise our curriculum such that it remains aligned with best practices for patient safety.

In our experience, to effectively conduct the practical portion of the scrub training session, the student-instructor ratio should not exceed eight to one. This allows for more personalized, learner-specific attention and guidance. We have also found that administering the knowledge assessment electronically enabled the provision of instantaneous feedback (in the form of a numerical score without divulging missed questions) and potentially reduced the number of learners simply memorizing correct/incorrect answers, as all questions can be successfully answered with close review of the scrub training video. For institutions considering implementing such a program without readily available donated items, we have found that the use of reusable gowns and reusing sterile gloves for assisting gowning and gloving was a viable solution for small groups. However, this is not recommended for large groups that necessitate the ability to self-gown and glove.

The acquisition of knowledge and skills in aseptic technique is a requisite for any provider in the medical field. We share this multimodal scrub training curriculum, mapped to the AAMC's EPA guidelines,[2,3] to reduce variability in teaching strategies and skills acquisition through a standardized curriculum. Also, we hoped to effectively impart these skills and instill a sense of confidence in learners as they work to provide their best in patient care and safety.

Appendices

A. Instructor Guide.docx
B. Scrub Training Video.mp4
C. Student Instructional Letter Template.docx
D. Scrub Training Knowledge Test.docx

Copyright © 2021 Anderson et al. This is an open-access publication distributed under the terms of the Creative Commons Attribution license.

E. Scrub Training Skills Checklist.docx

F. Scrub Training Pre- and Postsession Survey.docx

All appendices are peer reviewed as integral parts of the Original Publication.

Tiffany N. Anderson, MD, MHPE: Surgical Education Fellow, Goodman Surgical Education Center, Department of Surgery, Stanford University; ORCID: https://orcid.org/0000-0001-8471-1157

Brittany N. Hasty, MD, MHPE: Surgical Education Fellow, Goodman Surgical Education Center, Department of Surgery, Stanford University School of Medicine

Ingrid S. Schmiederer, MD: Surgical Education Fellow, Goodman Surgical Education Center, Department of Surgery, Stanford University School of Medicine

Sarah E. Miller, MD: Resident, Department of Obstetrics and Gynecology, Stanford University School of Medicine

Robert Shi, MS: Research Assistant, Goodman Surgical Education Center, Stanford University School of Medicine

Lauren R. Aalami: Research Assistant, Goodman Surgical Education Center, Stanford University School of Medicine

Elizabeth M. Huffman, MD: Surgical Education Fellow, Department of Surgery, Indiana University School of Medicine

Jennifer N. Choi, MD: Clinical Professor, General Surgery Residency Program Director, Department of Surgery, Indiana University School of Medicine

James N. Lau, MD, MHPE: Clinical Professor, Department of Surgery, Stanford University School of Medicine; Director, Goodman Surgical Education Center, Stanford University School of Medicine

Acknowledgments
We would like to thank Susan Gates for her many years of dedication to education and patient safety and those who have volunteered their time to these educational sessions.

Disclosures
None to report.

Funding/Support
This project was funded in part by the Stanford University School of Medicine's Teaching and Mentoring Academy Innovation Grant.

Ethical Approval
Stanford University School of Medicine's Institutional Review Board approved this study.

References

1. Leeper K, Stegall MS, Stegall MH. Basic aseptic technique for medical students: identifying essential entry-level competencies. *Curr Surg*. 2002;59(1):69-73. https://doi.org/10.1016/S0149-7944(01)00494-9
2. Amiel J, Emery M, Hormann M. EPA 12: Perform general procedures of a physician. In: Obeso V, Brown D, Phillipi C, eds. *Core Entrustable Professional Activities for Entering Residency: Toolkits for the 13 Core EPAs - Abridged*. Association of American Medical Colleges; 2017:16. Accessed November 29, 2020. https://www.aamc.org/system/files/c/2/484778-epa13toolkit.pdf
3. Crowe R, Hyderi A, Rosenfeld M, Uthman M, Yingling S. EPA 13: Identify system failures and contribute to a culture of safety and improvement. In: Obeso V, Brown D, Phillipi C, eds. *Core Entrustable Professional Activities for Entering Residency: Toolkits for the 13 Core EPAs - Abridged*. Association of American Medical Colleges; 2017:17. Accessed November 29, 2020. https://www.aamc.org/system/files/c/2/484778-epa13toolkit.pdf
4. Ravindra P, Fitzgerald JEF, Bhangu A, Maxwell-Armstrong CA. Quantifying factors influencing operating theater teaching, participation, and learning opportunities for medical students in surgery. *J Surg Educ*. 2013;70(4):495-501. https://doi.org/10.1016/j.jsurg.2013.02.011
5. Pettitt BJ. Medical student concerns and fears before their third-year surgical clerkship. *Am J Surg*. 2005;189(4):492-496. https://doi.org/10.1016/j.amjsurg.2004.09.017
6. Chapman SJ, Hakeem AR, Marangoni G, Raj Prasad K. How can we enhance undergraduate medical training in the operating room? A survey of student attitudes and opinions. *J Surg Educ*. 2013;70(3):326-333. https://doi.org/10.1016/j.jsurg.2013.01.008
7. Fernando N, McAdam T, Youngson G, McKenzie H, Cleland J, Yule S. Undergraduate medical students' perceptions and expectations of theatre-based learning: how can we improve the student learning experience? *Surgeon*. 2007;5(5):271-274. https://doi.org/10.1016/S1479-666X(07)80024-2
8. Fernando N, McAdam T, Cleland J, Yule S, McKenzie H, Youngson G. How can we prepare medical students for theatre-based learning? *Med Educ*. 2007;41(10):968-974. https://doi.org/10.1111/j.1365-2923.2007.02839.x
9. Merkow RP, Ju MH, Chung JW, et al. Underlying reasons associated with hospital readmission following surgery in the United States. *JAMA*. 2015;313(5):483-495. https://doi.org/10.1001/jama.2014.18614
10. Shipper ES, Miller SE, Hasty BN, Merrell SB, Lin DT, Lau JN. Evaluation of a technical and nontechnical skills curriculum for students entering surgery. *J Surg Res*. 2017;219:92-97. https://doi.org/10.1016/j.jss.2017.05.105
11. Drolet BC, Sangisetty S, Mulvaney PM, Ryder BA, Cioffi WG. A mentorship-based preclinical elective increases exposure,

Copyright © 2021 Anderson et al. This is an open-access publication distributed under the terms of the Creative Commons Attribution license.

confidence, and interest in surgery. *Am J Surg*. 2014;207(2):179-186. https://doi.org/10.1016/j.amjsurg.2013.07.031

12. Antiel RM, Thompson SM, Camp CL, Thompson GB, Farley DR. Attracting students to surgical careers: preclinical surgical experience. *J Surg Educ*. 2012;69(3):301-305. https://doi.org/10.1016/j.jsurg.2011.10.001
13. Hasty BN, Lau JN, Tekian A, et al. Validity evidence for a knowledge assessment tool for a mastery learning scrub training curriculum. *Acad Med*. 2020;95(1):129-135. https://doi.org/10.1097/ACM.0000000000003007
14. Ericsson KA. Acquisition and maintenance of medical expertise: a perspective from the expert-performance approach with deliberate practice. *Acad Med*. 2015;90(11):1471-1486. https://doi.org/10.1097/ACM.0000000000000939
15. Bandura A. Guide for creating self-efficacy scales. In: Pajares F, Urdan T, eds. *Self-Efficacy Beliefs of Adolescents*. Information Age Publishing; 2006:307-338.

Received: May 2, 2020
Accepted: October 6, 2020
Published: February 1, 2021

Copyright © 2021 Anderson et al. This is an open-access publication distributed under the terms of the Creative Commons Attribution license.
