# Supplementary material for: A Generalizable Multimodal Scrub Training Curriculum in Surgical Sterile Technique
Source: MedEdPORTAL. 2021 Feb 1;17:11077. doi: 10.15766/mep_2374-8265.11077 (PMC7852343; doi:10.15766/mep_2374-8265.11077)
Supplement: Supplementary file 1 — Instructor Guide.docxScrub Training Video.mp4Student Instructional Letter Template.docxScrub Training Knowledge Test.docxScrub Training Skills Checklist.docxScrub Training Pre- and Postsession Survey.docx [file mep_2374-8265.11077-s001.zip › A. Instructor Guide.docx]

**Instructor Guide:**

The purpose of this course is to provide a structured immersive *in situ* training experience that will positively influence the learners’ abilities in donning operating room (OR) attire, utilizing scrub training techniques, and acting in accordance with OR etiquette. This course should be appropriate for any learner requiring skills related to intraoperative participation requiring aseptic technique.

This guide should act as a blueprint to coordinate a session that can accommodate instructor to student ratio groups of up to 1:8. A medical background or OR experience is not absolutely mandatory to effectively facilitate this course; however, it is highly recommended. Consider enlisting instructors who are OR educators, surgical trainees, and/or surgical faculty. All instructors should successfully complete the “knowledge assessment” (with a 100% passing score – Appendix C) and demonstrate their ability to perform all skills listed on the scrub training “skills checklist” (Appendix D) independently. They should also be oriented to all of their institution’s pertinent OR policies.

Due to the *in situ* nature of this course it is recommended that the planned day of the session be coordinated with appropriate OR administration and management at least 1-2 months in advance to ensure the ability of the facility to accommodate the session. This course has also been successfully performed in a simulated environment. It is recommended that an early morning time is arranged (as to not interfere with scheduled cases). See Figure 1 for a suggested flow of activity.

Recommended equipment and material for all students:

Institutional specific operating room clothing (scrubs)

1 disposable surgical cap (bouffant, surgeons cap, or beard cap)

1 surgical mask with attached visor for eye protection

1 sterile surgical gown in sterile wrapping

1 pair of size appropriate surgical gloves

1 pair of size appropriate indicator surgical gloves

1 surgical scrub brush

1 sterile hand towel

OR tables to allow at least 2.5m^2^ of surface area for each learner

Appropriate waste disposal receptacles

On the day of the session all instructors who will be present in the OR suite should have institution specific operating room clothing (scrubs), appropriate head covering, and closed toed shoes. They should arrive at least 15-20 minutes prior to the start of the session to help with setup and student check-in.

It is also recommended that learners have a point of contact to guide them to the appropriate resources. Consider the use of a templated letter detailing instructions for the learners (Appendix F). This should ideally be provided to the learners no less than 24 hours prior to the session.

The overall course objectives are as follows.

**Educational Objectives**

By the end of this activity, learners will be able to:

1. Describe appropriate surgery attire for operating room personnel.
2. Identify all personal protection equipment necessary for entry into the operating room suite.
3. Demonstrate effectively and independently proper surgical hygiene, donning of a mask, gowning and gloving.
4. Describe and demonstrate understanding of aseptic techniques principles.

All instructors should be well versed with the scrub training video (Appendix A) and “knowledge assessment.” The video offers a frame for the structure of the hands-on session and it is not uncommon for learners to ask questions related to the video or assessment.

The video is approximately 16 minutes in length and details all aspects of the scrub training process as well as general OR etiquette. All information is mapped to the objectives. The knowledge assessment can be administered in electronic format (recommended) or in paper form. A passing score of >92% should be achieved prior to course participation. If the paper format is provided, consider extending the course by 20-30 minutes to allow for adequate time for completion. If the learner is unable to complete the assessment within the allotted time, consideration may be given for post-session completion.

Figure 1

Check-in

Check-in consists of attendance confirmation, verification of appropriate attire of the learner, completion of the knowledge assessment, and assignment to instructor group. This should be performed prior to entering the OR suite. Learners should also be asked to complete the pre-session survey (Appendix E) during this time.

OR Orientation

Orientation should begin prior to entering the OR suite. Consider having a single instructor give the initial introduction. This introduction should consist of identifying all instructors and verbally framing the plan of activities.

Example:

*Good morning everyone! Thank you all for arriving on time and getting checked in. As an introduction I am [insert name] and my colleagues are [insert names].*

*The plan for today is that before we go down to the operating rooms, we will review the OR dress code policy. And then, how to select and don the appropriate head covering and surgical mask.*

*We will then go to the OR suite where we will show you where the case schedule board and operating rooms are located.*

*From there you will break-up into your assigned groups and head into the operating room to practice how to perform a surgical scrub, to gown and glove with and without assistance, and to practice maintaining sterile technique.*

*This is a safe environment and it is okay to make mistakes. We want you to feel free to ask questions at any time and to hone your skills.*

*Welcome to scrub training! Let’s get started.*

The initial discussion should include review of OR and institutional policies such as dress code.

Also, during this period head coverings and surgical masks should be distributed. The main spokesperson should demonstrate to the learners how to don these two items. The learners should demonstrate the activity and instructors should circulate throughout the room correcting infractions. Learners should not be transitioned to the OR suite without appropriate demonstration of these two skills.

Afterward the learners can be escorted to the OR suite where they should be oriented to important locations (such as OR schedule, front desk, and operating rooms). The learners can then be split into their previously assigned group and taken to operating rooms for the scrub practicum session.

Scrubbing Practicum

The scrubbing practicum should take place in an OR room that is large enough to accommodate all learners. We do not recommend more than 25 learners to a room to reduce crowding. Each learner should be stationed at a flat surface, at approximately waist height, that allows for adequate space to arrange gown and gloves without contamination (approximately 2.5m^2^). A semi-circular formation of the learners’ position to the instructor allows for easy visualization of the instructor by the students and vice versa. Learners should be oriented to the environment of the room, such as how to enter, introducing oneself, were to find material, general location of sterile field.

The instructor should demonstrate all activities prior to having the students demonstrate. Instructors should consider having several “skills checklists” as cognitive aids for self and learners.

Learners should be distributed unopened sterile gowns and two sets of size appropriate standard sterile gloves and one set of size appropriate indicator glove. This is an ideal time for learners to try different sizes to ensure the best fit.

Tips for glove sizing: stated size is approximately equivalent to ring size and indicator glove is typically one-half size larger than the stated glove.

For details on how to perform each activity please refer to the video.

All learners should be allowed ample opportunity to practice each skill as their ability requires and time allows. Ideally, more time should be spent honing skills that will most often be used at a given institution. At the end of the session ALL learners must be able to demonstrate recognition of sterile field and opportunities for contamination.

Debrief

The debriefing portion of the session should be allotted for final questions, clean-up, completion of the “post-session survey” (Appendix E). Learners should be escorted from the OR suite as soon as all practice is complete. This allows for clean-up of the OR and progression of the normal scheduled day. As a parting note, learners should be encouraged to review the video as needed and refer to the skills cognitive aide to refresh their memory of expected skills and techniques within the intraoperative environment.

If a simulated environment is used every effort should be made to interact with the environment as though it were *in situ*.

Additional Discussion Points

Instructors should be well aware of gaps in the video that are reflective of their local environments. The following are example items that may be institution dependent that should be discussed with learners as appropriate.

 - Use of cap type:

-Bouffant only versus (vs.) skull cap vs. beard covering

-Cloth vs. disposable cap

 - Use of mask types:

  -Eye protection requirements (e.g. side shields for glasses vs. shield masks)

 - Use of gown types:

-Cloth gowns (modification in turning since a sterile person is required)

- Use of glove types:

-Availability of Biogel^®^ indicator gloves vs. double gloving with regular gloves

- Use of alternative Scrub Solutions:

  -Chlorhexidine vs. Iodine bases

-Alcohol (waterless) based solutions
