# Supplementary material for: A Generalizable Multimodal Scrub Training Curriculum in Surgical Sterile Technique
Source: MedEdPORTAL. 2021 Feb 1;17:11077. doi: 10.15766/mep_2374-8265.11077 (PMC7852343; doi:10.15766/mep_2374-8265.11077)
Supplement: Supplementary file 1 — Instructor Guide.docxScrub Training Video.mp4Student Instructional Letter Template.docxScrub Training Knowledge Test.docxScrub Training Skills Checklist.docxScrub Training Pre- and Postsession Survey.docx [file mep_2374-8265.11077-s001.zip › C. Student Instructional Letter Template.docx]

[Institutional Name]

[Applicable Locations]

Scrub Training Protocol

We are excited to have you coming to care for patient with [Institutional Name]. We, at [Institutional Name], place an overarching emphasis on patient safety and comprehensive patient care. It is in that vein that **you must adhere** to our patient safety scrub and sterile procedure training and protocol as you care for patients at [Institutional Name] in our Operative Areas.

**Prior to being allowed entry and participation (scrubbing in on cases) you must complete a Scrub and Sterile Procedure Training with a knowledge and skills assessment to proficiency.**

For training you are to report to [Location] at [Time]. It will be expected that you have already watched the scrub training video.

The link for the mandatory training video is: [Video Source]. As part of the overall curriculum, you are required to complete a knowledge assessment. This quiz is related to the material featured in the training video. A passing score of 92% is required.

(if electronically distributed) Knowledge assessment: [Online Source]

At [Time] you will be assessed on your OR attire, hand scrubbing, and sterile gowning and gloving technique. The assessment process, depending on how many other students require this, may take until [Time]. Depending on your performance this may extend your session.

We hope you understand that this process is to ensure sterility and infection prevention for our precious patients at [Institutional Name]. Thank you for your affability and understanding by complying with this important protocol.

Questions: [Primary Contact] (email/phone) and/or [Secondary Contact] (email/phone)
