# Supplementary material for: A Generalizable Multimodal Scrub Training Curriculum in Surgical Sterile Technique
Source: MedEdPORTAL. 2021 Feb 1;17:11077. doi: 10.15766/mep_2374-8265.11077 (PMC7852343; doi:10.15766/mep_2374-8265.11077)
Supplement: Supplementary file 1 — Instructor Guide.docxScrub Training Video.mp4Student Instructional Letter Template.docxScrub Training Knowledge Test.docxScrub Training Skills Checklist.docxScrub Training Pre- and Postsession Survey.docx [file mep_2374-8265.11077-s001.zip › D. Scrub Training Knowledge Test.docx]

| Item# | Item Content |
| --- | --- |
| 1. | Where should your identification badge be worn on your scrubs while in the operating room? a. On your waist   1. On the scrub top neckline* 2. It doesn’t matter |
| 2. | Which of the following is prohibited in the operating room?   1. Unscented perfume/cologne 2. Unscented deodorant 3. Earrings that extend below the ear lobe* 4. Closed toed shoes |
| 3. | Under what conditions can you wear a necklace in the operating room?   1. As long as it is “form-fitting” around the neck 2. As long as it can be concealed underneath scrubs 3. No jewelry should be worn around the neck in the operating room while scrubbed in* |
| 4. | In addition to hospital scrubs and closed toed shoes, what else should you wear prior to entering the perioperative area (ex. hallways and operating room front desk)?   1. Wear a face mask 2. Wear a scrub cap* 3. Don gloves |
| 5. | Upon entering the operating room you should be wearing:   1. Closed toed shoes and hospital scrubs 2. Closed toed shoes, hospital scrubs, scrub cap, and face mask* 3. Closed toed shoes, hospital scrubs, and scrub cap 4. Closed toed shoes, hospital scrubs, and face mask |
| 6. | Individuals with full beards must wear the following in the operating room: a. Standard mask   1. Special mask that provides full beard coverage* 2. N95 respirator mask 3. Full beards are not permitted in the operating room and must be shaved |
| 7. | Upon entering the operating room, you should do the following:  a. Introduce yourself, write your name on the board, pull your gown and gloves, and ask to help with set up*  b. Stand quietly and wait to be spoken to   1. Write your name on the board and stand quietly in the corner 2. Introduce yourself and pull your gown and gloves |
| 8. | While performing envelope opening of the surgical gown, the first tab of the blue drape should be opened in which direction?   1. Away from you * 2. Towards you 3. To the left 4. To the right |
| 9. | The individual who must document every person who enters the operating room is the: a. Circulating nurse*   1. Scrub tech 2. Charge nurse 3. Operating room assistant |
| 10. | A useful event that happens in the operating room that is a good indicator for when it is an appropriate time to scrub in is:   1. Surgical “time out” 2. Patient intubation 3. Preoperative foley placement 4. Patient skin preparation* |
| 11. | While performing the surgical scrub, your hands should be kept in which position relative to your elbows: a. Above your elbows*  b. Below your elbows |

|  | c. Parallel to your elbows | |
| --- | --- | --- |
| 12. | The surgical hand wash should begin distally at the fingertips and progress proximally to the level of the:  a. Wrist   1. Distal forearm 2. Proximal forearm 3. Two inches above the elbow* | |
| 13. | When self-gloving, what should be the orientation of the glove as you place it on your hand in preparation of putting it on?   1. Glove thumb facing down on top of your thumb* 2. Glove thumb facing up on top of your thumb 3. Glove thumb facing down on top of your pinky 4. Glove thumb facing up on top of your pinky | |
| 14. | When assisted-gloving with a scrub technician or scrub nurse, the convention for the order in which you don your gloves is:   1. Right hand indicator glove - right hand top glove - left hand indicator glove - left hand top glove 2. Right hand indicator glove - left hand indicator glove - right hand top glove - left hand top glove* 3. Right hand indicator glove - left hand indicator glove - left hand top glove - right hand top glove 4. Left hand indicator glove - right hand indicator glove - left hand top glove - right hand top glove | |
| 15. | In order to secure the final tie on the left side of your sterile gown, which direction should you spin?  a. Clockwise   1. Counterclockwise* 2. Either way will work | |
| 16. | Once you are gowned and gloved, your hands must be kept:   1. Below your waist 2. Above your shoulders 3. Anterior to the midaxillary line* | |
| 17. | By convention, which color implies sterility (ex. sterile drape, sterile gown)?  a. Blue*   1. Orange 2. Red 3. White | |
| 18. | If you contaminate yourself (become unsterile), or are told you have contaminated yourself by operating room staff, what should you do?   1. Remain quiet, finish what you were doing, step away from the operative field, and go re-gown and re-glove 2. Stop what you are doing, announce that you are contaminated, and back away from the operative field to go re-gown and re-glove* 3. Debate whether you are contaminated and if you really need to go re-gown and re-glove | |
| 19. | Once you have donned your blue indicator surgical gloves, you can don your stated surgical glove (outer glove) in what fashion:   1. Glove thumb facing down on top of your thumb 2. In the normal fashion as one would with a unsterile pair of gloves* 3. Glove thumb facing down on top of your pinky | |
| 20. | When navigating the hallways outside the operating rooms, a surgical MASK must be worn:  a. True  b. False* | |
| 21. | When navigating the hallways outside the operating rooms, a surgical CAP must be worn:  a. True*  b. False | |
| 22. | To enter the operating room, surgical shoe covers must be worn:  a. True  b. False* | |
| 23. | When opening and handing off your surgical gown to the scrub tech/nurse, do you yourself need to be sterile in order to present the gown? | |
|  | 1. Yes 2. No* | |
| 24. | A sterile person (someone in sterile gown and gloves) should assist you in securing the back of your sterile gown.  a. True  b. False* | |
| 25. | You can secure the final tie of your sterile gown by spinning with either a sterile or unsterile operating room staff member.  a. True* | |
|  | b. | False |

*Denotes the correct response
