# Supplementary material for: A Generalizable Multimodal Scrub Training Curriculum in Surgical Sterile Technique
Source: MedEdPORTAL. 2021 Feb 1;17:11077. doi: 10.15766/mep_2374-8265.11077 (PMC7852343; doi:10.15766/mep_2374-8265.11077)
Supplement: Supplementary file 1 — Instructor Guide.docxScrub Training Video.mp4Student Instructional Letter Template.docxScrub Training Knowledge Test.docxScrub Training Skills Checklist.docxScrub Training Pre- and Postsession Survey.docx [file mep_2374-8265.11077-s001.zip › E. Scrub Training Skills Checklist.docx]

**Surgical Scrubbing Procedure Checklist for Medical Students**

Check **Done** or **Not Done**

(May be used as a cognitive aid or for assessment purposes)

|  |  | **Done** | **Not Done** |
| --- | --- | --- | --- |
| 1 | Dons cap (bouffant or surgeons cap), If beard is present, places appropriate beard coverage, shoe covers (optional). |  |  |
| 2 | Ties mask at the crown of the head and the nape of the neck |  |  |
| 3 | Follows hospital policy regarding fingernail polish/jewelry/tattoos/perfume/scented lotions. |  |  |
| 4 | Open gown by first peeling tab away from self |  |  |
| 5 | Opens gloves onto sterile field |  |  |
| 6 | Removes debris from under nails, using nail pick under running water. |  |  |
| 7 | Using cleansing agent (chlorhexidine or betadine) cleans around all fingers in a circumferential manner, minimum of 5 scrubs per digit |  |  |
| 8 | Arms lathered to 2 inches proximal of the elbow, minimum 20 scrubs on all surface area |  |  |
| 9 | Rinses hands and arms, from fingertips to elbows, keeping fingertips higher than elbows at all times. |  |  |
| 10 | Keeps hands elevated, extended at the level between the shoulders and the hips |  |  |
| 11 | Accepts towel with extended right hand and dries left hand without contaminating the free end |  |  |
| 12 | Grabs underside of towel with left hand and dries the right hand without contaminating the free end |  |  |
| **Assisted Gowning and Gloving** | | | |
| 13 | Steps forward to accept awaiting gown, placing arms through sleeves |  |  |
| 14 | Fingers remain within the confines of gown sleeves |  |  |
| 15 | Inserts right hand into indicator glove |  |  |
| 16 | Extends gloved right hand and assists with insertion of left hand into left glove |  |  |
| 17 | Assists with second set of gloves |  |  |
| 18 | Extends gown card on the longer tie |  |  |
| 19 | Spins in a counterclockwise direction and ties ends |  |  |
| **Self-Gowning and Gloving** | | | |
| 20 | Slides both arms into the sleeves without extending hands through the cuffs and keeps the sleeves of the gown above waist level and unravels the gown |  |  |
| 21 | Fingers remain in confines of sleeves |  |  |
| 22 | Places right glove with palm down and thumb aligned with own thumb |  |  |
| 23 | Places left glove with assistance from right hand |  |  |
| 24 | Puts on top layer of gloves (no special technique) |  |  |
| 25 | Extends gown card on the longer tie |  |  |
| 26 | Spins in a counterclockwise direction and ties ends |  |  |

**Recommendation: Pass ­­­­____ Needs more practice _____**

**Instructor: _______________________________ Date: __________________**
