# Supplementary material for: A Generalizable Multimodal Scrub Training Curriculum in Surgical Sterile Technique
Source: MedEdPORTAL. 2021 Feb 1;17:11077. doi: 10.15766/mep_2374-8265.11077 (PMC7852343; doi:10.15766/mep_2374-8265.11077)
Supplement: Supplementary file 1 — Instructor Guide.docxScrub Training Video.mp4Student Instructional Letter Template.docxScrub Training Knowledge Test.docxScrub Training Skills Checklist.docxScrub Training Pre- and Postsession Survey.docx [file mep_2374-8265.11077-s001.zip › F. Scrub Training Pre- and Postsession Survey.docx]

**Scrub Training Presession Survey**

|  | **Not able** | **Poor** | **Fair** | **Moderate** | **Excellent** |
| --- | --- | --- | --- | --- | --- |
| What is your current ability to correctly describe appropriate attire operating room personnel? |  |  |  |  |  |
| What is your current ability to correctly identify all personal protection equipment necessary for entry into the operating room suite? |  |  |  |  |  |
| What is your ability to appropriately perform a surgical hygiene “scrub”? |  |  |  |  |  |
| What is your ability to select and don the appropriate mask? |  |  |  |  |  |
| What is your ability to perform the assisted gowning and gloving technique without contamination? |  |  |  |  |  |
| What is your ability to perform the independent gowning and gloving technique without contamination? |  |  |  |  |  |
| What is your ability to identify sterile versus non-sterile surfaces within the operating suite? |  |  |  |  |  |
| What is your ability to identify sources of contamination within the operating suite? |  |  |  |  |  |

1. What aspect of the “scrubbing-in” process (washing, gowning, gloving, OR orientation, OR etiquette, etc…) causes you the **MOST** anxiety?
2. What aspect of the “scrubbing-in” process (washing, gowning, gloving, OR orientation, OR etiquette, etc…) causes you the **LEAST** anxiety?
3. What aspect of the “scrubbing-in” process (washing, gowning, gloving, OR orientation, OR etiquette, etc…) should we spend more time on?

Have you taken a formal scrub training course before?

Yes

No

Have you ever scrubbed into a surgical case before?

Yes

No

**Scrub Training Postsession Survey**

|  | **Not able** | **Poor** | **Fair** | **Moderate** | **Excellent** |
| --- | --- | --- | --- | --- | --- |
| What is your current ability to correctly describe appropriate attire operating room personnel? |  |  |  |  |  |
| What is your current ability to correctly identify all personal protection equipment necessary for entry into the operating room suite? |  |  |  |  |  |
| What is your ability to appropriately perform a surgical hygiene “scrub”? |  |  |  |  |  |
| What is your ability to select and don the appropriate mask? |  |  |  |  |  |
| What is your ability to perform the assisted gowning and gloving technique without contamination? |  |  |  |  |  |
| What is your ability to perform the independent gowning and gloving technique without contamination? |  |  |  |  |  |
| What is your ability to identify sterile versus non-sterile surfaces within the operating suite? |  |  |  |  |  |
| What is your ability to identify sources of contamination within the operating suite? |  |  |  |  |  |

1. What aspect of the “scrubbing-in” process (washing, gowning, gloving, OR orientation, OR etiquette, etc…) causes you the **MOST** anxiety?
2. What aspect of the “scrubbing-in” process (washing, gowning, gloving, OR orientation, OR etiquette, etc…) causes you the **LEAST** anxiety?
3. What aspect of the “scrubbing-in” process (washing, gowning, gloving, OR orientation, OR etiquette, etc…) should we spend more time on?
